# Supplementary material for: Resolving the full spectrum of human genome variation using Linked-Reads
Source: Genome Res. 2019 Apr;29(4):635–45. doi: 10.1101/gr.234443.118 (PMC6442396; doi:10.1101/gr.234443.118)
Supplement: Supplemental Material [file supp_gr.234443.118_Supplemental_longranger-2.2.zip › longranger-2.2.tar.bz2!/lr2.2-public/lib/stan/cmdstan/stan/doc/api/html/rev_2operators_8hpp.html]

Stan: src/stan/agrad/rev/operators.hpp File Reference


|  |  |
| --- | --- |
|  | Stan  2.5.0  probability, sampling & optimization |


- Main Page
- Related Pages
- Namespaces
- Classes
- Files

- File List
- File Members

All Classes Namespaces Files Functions Variables Typedefs Enumerations Enumerator Friends Macros Pages

- src
- stan
- agrad
- rev

operators.hpp File Reference

`#include <stan/agrad/rev/operators/operator_addition.hpp>`  
`#include <stan/agrad/rev/operators/operator_divide_equal.hpp>`  
`#include <stan/agrad/rev/operators/operator_division.hpp>`  
`#include <stan/agrad/rev/operators/operator_equal.hpp>`  
`#include <stan/agrad/rev/operators/operator_greater_than.hpp>`  
`#include <stan/agrad/rev/operators/operator_greater_than_or_equal.hpp>`  
`#include <stan/agrad/rev/operators/operator_less_than.hpp>`  
`#include <stan/agrad/rev/operators/operator_less_than_or_equal.hpp>`  
`#include <stan/agrad/rev/operators/operator_minus_equal.hpp>`  
`#include <stan/agrad/rev/operators/operator_multiplication.hpp>`  
`#include <stan/agrad/rev/operators/operator_multiply_equal.hpp>`  
`#include <stan/agrad/rev/operators/operator_not_equal.hpp>`  
`#include <stan/agrad/rev/operators/operator_plus_equal.hpp>`  
`#include <stan/agrad/rev/operators/operator_subtraction.hpp>`  
`#include <stan/agrad/rev/operators/operator_unary_decrement.hpp>`  
`#include <stan/agrad/rev/operators/operator_unary_increment.hpp>`  
`#include <stan/agrad/rev/operators/operator_unary_negative.hpp>`  
`#include <stan/agrad/rev/operators/operator_unary_not.hpp>`  
`#include <stan/agrad/rev/operators/operator_unary_plus.hpp>`

Go to the source code of this file.


---

[ Stan Home Page ]

*© 2011–2014,
Stan Development Team.*
